# Comparative Cardio-Renal Outcomes of Type 2 Diabetes Patients Administered Glucagon-Like Peptide-1 Receptor Agonists: A Network Meta-Analysis

**DOI:** 10.3389/fphar.2021.759262

**Published:** 2021-12-24

**Authors:** Chuanjun Zhuo, Chongguang Lin, Chunhua Zhou, Xiangyang Gao, Hailin Shao, Tao Fang, Hongjun Tian, Li Ding, Ming Liu

**Affiliations:** ^1^ National of Metabolism Management Center (MMC), Tianjin Medical University Affiliated Tianjin Fourth Center Hospital, Nankai University Affiliated Hospital, Tianjin Fourth Center Hospital, Tianjin, China; ^2^ Department of Endocrinology and Metabolism, Tianjin Medical University General Hospital, Tianjin, China; ^3^ Department of Psychiatric-Neuroimaging-Genetics Laboratory (PNGC_Lab), Tianjin Mental Health Center, Tianjin Medical University, Tianjin, China; ^4^ Department of Psychiatry, Wenzhou Seventh Peoples Hospital, Wenzhou, China; ^5^ Department of Pharmacy, The First Hospital of Hebei Medical University, Shijiazhuang, China; ^6^ Big Data Analysis Center of Health Management Institute, The Second Medical Center and National Clinical Research Center for Geriatric Diseases, Chinese PLA General Hospital, Beijing, China

**Keywords:** cardio-renal benefit, glucagon-like peptide-1 receptor agonist, network meta-analysis, semaglutide, type 2 diabetes

## Abstract

**Background:** Cardio-renal profiles are available from cardiovascular outcome trials of glucagon-like peptide-1 receptor agonists (GLP-1 RAs).

**Methods:** A comprehensive systematic review of Embase, Medline, Web of Knowledge, and CENTRAL databases was conducted. Randomized controlled cardiovascular outcome trials of type 2 diabetes mellitus (T2DM) patients administered GLP-1 RAs were included. The following primary outcomes were examined: cardiovascular death, major adverse cardiovascular events (MACE), myocardial infarction, stroke, mortality, heart failure, hypoglycemia, pancreatitis, and thyroid carcinoma. Secondary outcomes included: composite kidney outcome, worsening kidney function, macroalbuminuria, and retinopathy.

**Results:** Seven trials involving 56,004 patients and eight interventions were identified. Albiglutide was associated with fewer MACE and myocardial infarction events compared with lixisenatide. Lixisenatide was related to a greater number of stroke events and cardiovascular deaths compared to once-weekly semaglutide and oral semaglutide, respectively. Improved mortality was associated with oral semaglutide compared with once-weekly semaglutide, albiglutide, dulaglutide, exenatide, or lixisenatide. Risks of heart failure, thyroid carcinoma, and pancreatitis were similar among all the treatments. Weighting of the nine primary outcomes identified oral semaglutide as first among the eight treatments examined. Among three of the secondary outcomes, once-weekly semaglutide ranked first. Better composite kidney outcome was observed with once-weekly semaglutide than with dulaglutide or exenatide; once-weekly semaglutide improved macroalbuminuria compared with exenatide or lixisenatide; and albiglutide, exenatide, and placebo was associated with fewer cases of retinopathy compared with once-weekly semaglutide. Meanwhile, kidney function was less likely to worsen with dulaglutide than with lixisenatide or placebo.

**Conclusion:** Semaglutide should be considered when GLP-1 RAs are indicated for T2DM patients.

## Introduction

Type 2 diabetes (T2DM) is a prevalent illness that causes major cardiovascular, renal, and neurologic complications (Chatterjee et al., 2017). Use of traditional antidiabetic drugs to control blood glucose has reduced the numbers of micro-vascular events in T2DM patients, yet has not been able to significantly reduce the risk of macro-vascular events in all patients (Group UPDSU, 1998; Action to Control Cardiovascular Risk in Diabetes Study Group et al., 2008; Holman et al., 2008; Control Group et al., 2009; Gerstein et al., 2014; Hayward et al., 2015; Wong et al., 2016). Furthermore, concerns about myocardial infarction and greater risk of heart failure that are associated with effective hypoglycemic agents, such as pioglitazone and rosiglitazone, have led to greater awareness of potentially harmful cardiovascular effects (Home et al., 2007; Lago et al., 2007; Nissen and Wolski 2007). Therefore, in 2008, the U.S. Food and Drug Administration (FDA) started providing regulatory guidance for industry mandated cardiovascular outcome trials (CVOTs) to evaluate novel antidiabetic agents and cardiovascular safety (Wilcox et al., 2020). The FDA has also provided evidence that hemoglobin A1c (HbA1C) reduction does not significantly reduce cardiovascular morbidity or mortality (Wilcox et al., 2020).

Over the past 10 years, the number of CVOTs for new glucose-lowering medications which target numerous novel pathways has been steadily increasing. Early CVOTs included patients with pre-existing cardiovascular diseases, while later CVOTs have included populations at high risk of cardiovascular disease (Del Olmo-Garcia and Merino-Torres, 2018; Acharya and Deedwania 2019). Initially, these CVOTs were designed to evaluate the safety of glucose-lowering medications. However, trials of sodium-glucose co-transporter 2 inhibitors (SGLT2i) and glucagon-like peptide-1 receptor agonists (GLP-1 RAs) have demonstrated cardiovascular benefits in secondary prevention of major adverse cardiovascular events (MACE), including cardiovascular death, heart failure, stroke, and myocardial infarction (Vijan 2019). Therefore, the American Diabetes Association (ADA) and the European Association for the Study of Diabetes (EASD), while previously employing a purely glucocentric approach for T2DM, have recently adopted a more holistic strategy with use of agents that demonstrate cardiorenal superiority (Davies et al., 2018; Cosentino et al., 2020). This recommendation reflects the wealth of clinical trial evidence regarding the health benefits associated with GLP-1RAs and SGLT2i.

GLP-1 is predominantly produced in enteroendocrine cells and it coordinates energy homeostasis and glucose metabolism via regulation of food intake, islet hormone secretion, and gastrointestinal motility. Consequently, GLP-1 RAs have been developed for the treatment of T2DM and obesity (Drucker 2016). In general, CVOTs of GLP-1 RAs have included randomized patients with T2DM in active therapy or placebo groups. These studies have investigated whether specific GLP-1 RAs improve cardiovascular and renal outcomes. To date, two studies have tried to synthesize these complex data. In one meta-analysis study, GLP-1 RAs were shown to provide beneficial effects on mortality, kidney, and cardiovascular outcomes in patients with T2DM (Kristensen et al., 2019). In a second network meta-analysis, mortality and cardiovascular safety were indirectly compared among various GLP-1 RAs in T2DM patients (Alfayez et al., 2020). However, to date, there remains no clear evidence to indicate which GLP-1 RA is optimal in terms of cardio-renal and other key outcomes. Therefore, the objective of the present study was to conduct a network meta-analysis with a systematic review of all COVTs of GLP-1 RAs in order to: (1) compare the primary outcomes reported, including cardiovascular outcomes, hypoglycemia, pancreatitis, thyroid medullary, and papillary carcinoma, (2) compare the secondary outcomes reported in a subset of the CVOTs, including composite kidney outcome, worsening kidney function, macroalbuminuria, retinopathy, and (3) rank the integrative effect of GFP-1 RAs on both primary and secondary outcomes in T2DM patients.

## Methods

### Systematic Literature Search

According to the PRISMA Extension Statement for Reporting of Systematic Reviews Incorporating Network Meta-analyses of Health Care Interventions: Checklist and Explanations (Hutton et al., 2015), a network meta-analysis was conducted. The study protocol was prespecified and registered on International Platform of Registered Systematic Review and Meta-analysis (INPLASY) (registration number, INPLASY202080122). A systematic review was conducted to identify published CVOTs for GLP-1 RAs in the following databases from the inception of these databases up to August 1, 2020: Embase, Medline (via PubMed), Web of Knowledge, and Cochrane Central Register of Controlled Trials (CENTRAL). The following keywords and MeSH terms were used: glucagon like peptide-1, GLP-1 receptor agonist, lixisenatide, liraglutide, semaglutide, exenatide, albiglutide, dulaglutide, myocardial infarction, heart failure, death, mortality, stroke, and angina. References cited in identified articles and those in relevant meta-analyses and systematic reviews were examined to identify articles not identified in the computerized database search. There were no limitations on language or publication year for study selection.

### Study Selection

Randomized controlled trials which examined cardiovascular safety as a primary outcome for both injectable and oral GLP-1 RAs (at least two GLP-1 RAs or a GLP-1 RA and a placebo) administered to patients with T2DM were selected for the network meta-analysis conducted in this study (Supplementary Table S1). Conflicts regarding study inclusion were resolved by consensus.

### Data Extraction

Two authors independently extracted data from the selected published studies. Study design, characteristics of participants, treatments, follow-up, primary and secondary outcomes, cardiovascular disease, systolic blood pressure, history of heart failure, estimated glomerular filtration rate (eGFR), and use of other drugs were documented. Any disagreements regarding data extraction were resolved with a third investigator.

### Primary and Secondary Outcomes

The primary outcomes of interest included: non-fatal or fatal myocardial infarction, non-fatal or fatal stroke, MACE, cardiovascular death, all-cause mortality, hospital admission for heart failure, severe hypoglycemia, pancreatitis, and thyroid carcinoma. Secondary outcomes of interest included: composite kidney outcomes, worsening kidney function, macroalbuminuria, and retinopathy. Both primary and secondary outcomes are defined in Supplementary Table S2.

### Risk of Bias Assessment

For individual studies of randomized trials, the Cochrane risk of bias tool was applied by two independent reviewers (Higgins et al., 2011). Studies were classified as having low, high, or unclear risk of bias depending on allocation concealment, sequence generation adequacy, blinding of personnel and participants, as well as blinding of outcome assessment, selective reporting, and method of addressing incomplete data. Graphic representations of potential bias both across and within studies were generated with RevMan V.5.1. To resolve disagreements, discussion, then consultation of a third arbitrator, was employed, respectively.

### Statistical Analysis

To compare different GLP-1 RAs, a frequentist network meta-analysis was conducted by using the network command in STATA (White, 2009, 2011; Higgins et al., 2012; White et al., 2012). Evaluation of safety between at least two GLP-1 RAs, or between a GLP-1 RA and placebo, is reported with odds ratio (OR) values and corresponding 95% confidence intervals (CIs). It was assumed that a common heterogeneity parameter exists across all of the loops in the network. Therefore, inconsistency checks could be performed within a closed loop in the network (Higgins et al., 2003; Veroniki et al., 2013). A simple transformation of mean rank generated a surface under the cumulative ranking curve (SUCRA). As a result, a hierarchy of the treatments could be obtained and both the variance and location of all the relative treatment effects could be accounted for (Salanti et al., 2011). SUCRA values could vary from 100 to 0, representing the best to worst interventions, respectively. STATA version 14.0 software (Stata Corporation, College Station, TX, United States) was used to perform all of the statistical analyses described.

## Results

### Characteristics of the Studies Examined

**FIGURE 1:**
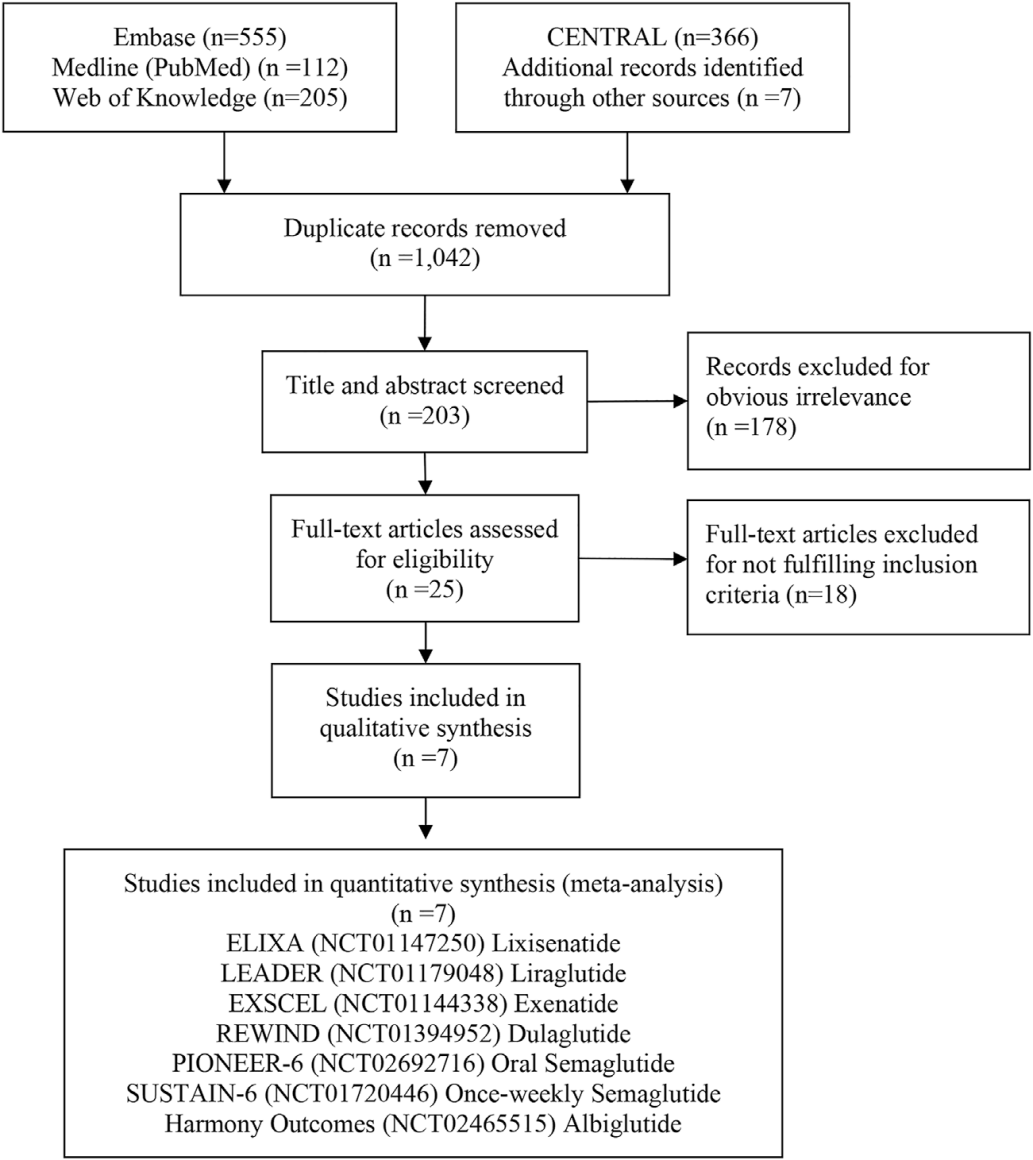
Schematic illustration of the literature search and study selection performed.

A total of 1,245 studies were retrieved from databases and relevant articles, of which seven met the inclusion criteria for analysis (Figure 1). These seven studies included a total of 56,004 T2DM patients who received the following treatments: lixisenatide (ELIXA) (Pfeffer et al., 2015; Muskiet et al., 2018), liraglutide (LEADER) (Mann et al., 2017), once-weekly semaglutide (SUSTAIN-6) (Marso et al., 2016), albiglutide (Harmony Outcomes) (Hernandez et al., 2018), exenatide (EXSCEL) (Holman et al., 2017), oral semaglutide (PIONEER 6) (Husain et al., 2019), and dulaglutide (REWIND) (Gerstein et al., 2019a,b). Lixisenatide and exenatide are short-acting exendin-4 compounds, while semaglutide, albiglutide, liraglutide, and dulaglutide are long-acting human GLP-1 compounds. All seven studies, published between 2015 and 2019, had sample sizes which ranged from 3,183 to 14,752 participants (Table 1).

**TABLE 1:** Characteristics of the studies included in the meta-analysis.

Study	ELIXA	LEADER	SUSTAIN-6	EXSCEL	HARMONY	REWIND	PIONEER-6
Trial no.	NCT01147250	NCT01179048	NCT01720446	NCT01144338	NCT02465515	NCT01394952	NCT02692716
Country	United States	United States, Germany	United States	United Kingdom	United Kingdom	Canada	Canada
Design	RCT	RCT	RCT	RCT	RCT	RCT	RCT
Drug	Lixisenatide	Liraglutide	Semaglutide	Exenatide	Albiglutide	Dulaglutide	Semaglutide
Structural basis	Exendin-4	Human GLP-1	Human GLP-1	Exendin-4	Human GLP-1	Human GLP-1	Human GLP-1
Action time	Short-acting	Long-acting	Long-acting	Short-acting	Long-acting	Long-acting	Long-acting
Administration route	Subcutaneous	Subcutaneous	Subcutaneous	Subcutaneous	Subcutaneous	Subcutaneous	Oral
Dose	20 μg/day	1.8 mg/day	0.5/1 mg/week	2 mg/week	30/50 mg/week	1.5 mg/week	14 mg/day
Medications	Once daily vs. placebo	Once daily vs. placebo	Once weekly vs. placebo	Once weekly vs. placebo	Once weekly vs. placebo	Once weekly vs. placebo	Once daily vs. placebo
Sample size, N	6,068	9,340	3,297	14,752	9,463	9,901	3,183
Median follow-up (y)	2.1	3.8	2.1	3.2	1.6	5.4	1.3
Age (y), Mean (SD)	59.9 (9.7)	64.2 (7.2)	64.6 (7.4)	62.7 (3.6)	64.1 (8.7)	66.2 (6.5)	66 (7.0)
Males, N (%)	4,207 (69)	6,003 (64)	2,002 (61)	9,148 (62)	6,569 (69)	5,312 (53.7)	2,176 (68.4)
Ethnic origin							
White, N (%)	4,576 (75)	7,238 (77)	2,736 (83)	11,175 (76)	6,583 (70)	7,498 (76)	2,300 (72)
Other, N (%)	1,492 (25)	2,102 (23)	561 (17)	3,577 (24)	2,880 (30)	2,403 (24)	883 (28)
BMI (kg/m^2^), Mean (SD)	30.1 (5.6)	32.5 (6.3)	32.8 (6.2)	32.7 (6.4)	32.3 (5.9)	32.3 (5.7)	32.3 (6.5)
Duration of diabetes (y), Mean (SD)	9.2 (8.2)	12.8 (8.0)	13.9 (8.1)	12.3 (3.2)	14.1 (8.6)	10.5 (7.3)	14.9 (8.5)
HbA1c (%), Mean (SD)	7.7 (1.3)	8.7 (1.6)	8.7 (1.5)	8.1 (0.5)	8.7 (1.5)	7.3 (1.1)	8.2 (1.6)
Existence of CVD, N (%)	6,068 (100)	7,598 (81)	2,735 (83)	10,782 (73)	6,678 (71)	3,114 (31)	2,695 (85)
History of heart failure, N (%)	1,358 (22)	1,667 (18)	777 (24)	2,389 (16)	1,922 (20)	853 (9)	388 (12)
Systolic blood pressure (mm Hg), Mean (SD)	129 (17)	136 (18)	136 (17)	135 (17)	135 (17)	137 (17)	136 (18)
eGFR (mL/min per 1·73 m^2^)*	78 (21)	80 (NR)	80 (61–92)	77 (61–92)	79 (25)	75 (24)	74 (21)
Statin use, N (%)	5,627 (92.7)	6,741 (72.2)	2,399 (72.8)	10,836 (73.5)	7,955 (84.1)	6,547 (66.1)	2,712 (85.2)
Insulin, N (%)	2,374 (39)	4,169 (45)	1,913 (58)	6,838 (46)	5,597 (59)	2,363 (24)	1,930 (61)
Biguanides, N (%)	4,021 (66)	7,144 (76)	2,414 (73)	11 ,295 (77)	6,969 (74)	8,037 (81)	2,463 (77)
Sulfonylurea, N (%)	2,004 (33)	4,733 (51)	1,410 (43)	5,401 (37)	2,725 (29)	4,552 (46)	1,027 (32)
Thiazolidinedione, N (%)	95 (2)	575 (6)	76 (2)	579 (4)	194 (2)	168 (2)	118 (4)
DPP-4 inhibitor, N (%)	NA	6 (<1)	5 (<1)	2,203 (15)	1,437 (15)	88 (1)	2 (<1)
SGLT2 inhibitor, N (%)	NA	NA	5 (<1)	77 (1)	575 (6)	12 (<1)	305 (10)

RCT, randomized, double-blind, placebo-controlled trial; SD, standard deviation; CVD, cardiovascular disease; eGFR, estimated glomerular filtration rate; NR, not reported; DPP-4, dipeptidyl peptidase 4; SGLT2, sodium-glucose co-transporter 2; NA, not available.

**FIGURE 2:**
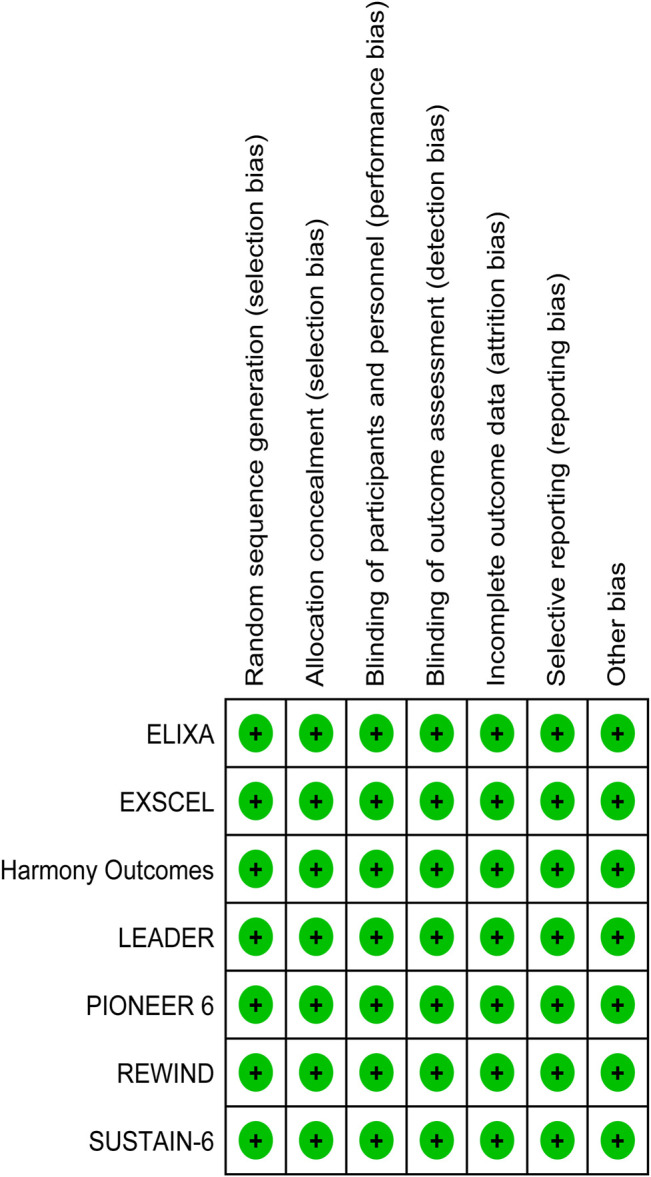
Risk of bias graph.

ELIXA (Pfeffer et al., 2015) included T2DM patients who recently experienced acute coronary syndrome, while the other six studies included T2DM patients with at least one co-existing cardiovascular condition or cardiovascular risk factor. The latter studies were conducted in the United Kingdom, Germany, United States, and Canada. The mean age of the participants ranged from 59.9 to 66.2 years, the proportion of male patients ranged from 53.7 to 69.0%, body mass index values ranged from 30.1 to 32.8, the mean duration of T2DM ranged from 9.2 to 11.9 years, and the median HbA1c value ranged from 7.7 to 8.7. The median duration of follow-up ranged from 1.3 years (PIONEER 6) to 5.4 years (REWIND). The proportion of patients with a history of heart failure or established cardiovascular disease ranged from 9% (REWIND) to 24% (SUSTAIN-6), or 31% (REWIND) to 100% (ELIXA), respectively. The REWIND, REWIND, ELIXA, and HARMONY trials reported the lowest use of statins (66.1%), insulin (24.0%), biguanides (66%), and sulfonylurea (29%) at baseline, respectively. Use of thiazolidinedione was similar across all seven trials, ranging from 2 to 6%. Values for eGFR ranged from 74 to 80 ml/min per m^2^ (Table 1). All seven studies were characterized as high quality with a lower risk of bias according to the Cochrane risk-of-bias tool (Figure 2).

### Network Meta-Analyses of Primary Outcomes

All seven trials reported all-cause mortality, MACE, hospital admittance for heart failure, severe hypoglycemia, pancreatitis, and thyroid carcinoma. Placebo was associated with a greater number of MACE compared with albiglutide (OR, 1.29; 95% CI: 1.11, 1.50), dulaglutide (OR, 1.13; 95% CI: 1.01, 1.28), liraglutide (OR, 1.16; 95% CI: 1.04, 1.31), and once-weekly semaglutide (OR, 1.39; 95% CI: 1.07, 1.79). Meanwhile, albiglutide was superior to lixisenatide (OR, 0.76; 95% CI: 0.61, 0.94) (Table 2).

**TABLE 2:** Network meta-analysis of nine primary outcomes.

Network meta-analysis of MACE (lower left) and fatal or non-fatal myocardial infarction (upper right)							
**Placebo**	**1.34 (1.10, 1.64)**	1.04 (0.86, 1.25)	1.02 (0.89, 1.16)	1.17 (1.00, 1.38)	0.96 (0.81, 1.15)	0.94 (0.59, 1.51)	1.38 (0.94, 2.02)
**1.29 (1.11, 1.50)**	**Albiglutide**	0.77 (0.59, 1.01)	**0.76 (0.60, 0.96)**	0.87 (0.68, 1.13)	**0.72 (0.55, 0.94)**	0.70 (0.42, 1.17)	1.02 (0.67, 1.58)
**1.13 (1.01, 1.28)**	0.88 (0.73, 1.06)	**Dulaglutide**	0.98 (0.78, 1.23)	1.13 (0.88, 1.45)	0.93 (0.72, 1.20)	0.91 (0.55, 1.51)	1.33 (0.87, 2.03)
1.08 (0.98, 1.20)	0.84 (0.70, 1.00)	0.96 (0.82, 1.12)	**Exenatide**	1.15 (0.94, 1.42)	0.95 (0.76, 1.18)	0.93 (0.57, 1.51)	1.35 (0.90, 2.03)
**1.16 (1.04, 1.31)**	0.90 (0.75, 1.09)	1.03 (0.87, 1.21)	1.08 (0.92, 1.26)	**Liraglutide**	0.82 (0.65, 1.05)	0.81 (0.49, 1.32)	1.17 (0.77, 1.78)
0.98 (0.84, 1.13)	**0.76 (0.61, 0.93)**	0.86 (0.71, 1.04)	0.90 (0.75, 1.08)	0.84 (0.69, 1.01)	**Lixisenatide**	0.98 (0.59, 1.62)	1.43 (0.94, 2.18)
1.26 (0.89, 1.77)	0.97 (0.67, 1.42)	1.11 (0.77, 1.60)	1.16 (0.81, 1.66)	1.08 (0.75, 1.55)	1.29 (0.88, 1.87)	**Oral Semaglutide**	1.46 (0.80, 2.67)
**1.39 (1.07, 1.79)**	1.07 (0.80, 1.44)	1.22 (0.92, 1.62)	1.28 (0.97, 1.69)	1.19 (0.89, 1.58)	1.42 (1.05, 1.91)	1.10 (0.72, 1.69)	**Once-weekly Semaglutide**
Network meta-analysis of fatal or non-fatal stroke (lower left) and cardiovascular death (upper right)							
**Placebo**	1.07 (0.83, 1.37)	1.10 (0.94, 1.29)	1.13 (0.97, 1.31)	**1.29 (1.07, 1.54)**	1.01 (0.81, 1.27)	**2.02 (1.08, 3.77)**	1.05 (0.69, 1.59)
1.15 (0.87, 1.52)	**Albiglutide**	1.03 (0.77, 1.38)	1.06 (0.79, 1.41)	1.20 (0.88, 1.64)	0.95 (0.68, 1.33)	1.89 (0.97, 3.70)	0.98 (0.60, 1.60)
**1.31 (1.06, 1.62)**	1.14 (0.80, 1.61)	**Dulaglutide**	1.03 (0.83, 1.28)	1.17 (0.92, 1.49)	0.92 (0.70, 1.22)	1.84 (0.97, 3.50)	0.95 (0.61, 1.49)
1.16 (0.96, 1.42)	1.01 (0.72, 1.42)	0.89 (0.67, 1.19)	**Exenatide**	1.14 (0.90, 1.44)	0.90 (0.69, 1.18)	1.79 (0.94, 3.40)	0.93 (0.59, 1.45)
1.16 (0.94, 1.42)	1.00 (0.71, 1.42)	0.88 (0.66, 1.19)	0.99 (0.75, 1.32)	**Liraglutide**	0.79 (0.59, 1.05)	1.57 (0.82, 3.01)	0.81 (0.52, 1.29)
0.89 (0.63, 1.27)	0.78 (0.49, 1.22)	0.68 (0.45, 1.03)	0.77 (0.51, 1.15)	0.77 (0.51, 1.16)	**Lixisenatide**	**1.99 (1.03, 3.87)**	1.03 (0.64, 1.66)
1.31 (0.63, 2.71)	1.14 (0.52, 2.47)	1.00 (0.47, 2.13)	1.13 (0.53, 2.39)	1.13 (0.53, 2.41)	1.47 (0.65, 3.28)	**Oral Semaglutide**	0.52 (0.24, 1.10)
**1.65 (1.01, 2.67)**	1.43 (0.82, 2.50)	1.26 (0.74, 2.13)	1.41 (0.84, 2.38)	1.42 (0.84, 2.41)	**1.84 (1.01, 3.35)**	1.26 (0.53, 3.00)	**Once-weekly Semaglutide**
Network meta-analysis of all-cause mortality (lower left) and hospital admission for heart failure (upper right)							
**Placebo**	1.17 (0.96, 1.42)	1.06 (0.88, 1.29)	1.05 (0.87, 1.27)	1.14 (0.95, 1.38)	1.04 (0.81, 1.34)	1.14 (0.63, 2.06)	0.91 (0.63, 1.33)
1.05 (0.86, 1.28)	**Albiglutide**	0.91 (0.69, 1.20)	0.90 (0.68, 1.18)	0.98 (0.75, 1.29)	0.89 (0.65, 1.23)	0.98 (0.53, 1.83)	0.78 (0.51, 1.20)
1.12 (0.99, 1.27)	1.07 (0.84, 1.35)	**Dulaglutide**	0.99 (0.76, 1.29)	1.08 (0.82, 1.41)	0.98 (0.71, 1.35)	1.08 (0.58, 2.00)	0.86 (0.56, 1.31)
**1.16 (1.02, 1.31)**	1.11 (0.87, 1.40)	1.04 (0.87, 1.23)	**Exenatide**	1.09 (0.84, 1.42)	0.99 (0.72, 1.36)	1.09 (0.59, 2.02)	0.87 (0.57, 1.32)
**1.19 (1.03, 1.37)**	1.14 (0.89, 1.45)	1.06 (0.88, 1.29)	1.03 (0.85, 1.24)	**Liraglutide**	0.91 (0.67, 1.25)	1.00 (0.54, 1.86)	0.80 (0.52, 1.21)
1.06 (0.87, 1.29)	1.01 (0.77, 1.34)	0.95 (0.75, 1.20)	0.92 (0.73, 1.15)	0.89 (0.70, 1.14)	**Lixisenatide**	1.10 (0.58, 2.09)	0.87 (0.56, 1.38)
**1.98 (1.19, 3.29)**	**1.89 (1.10, 3.27)**	**1.77 (1.05, 2.99)**	**1.71 (1.02, 2.89)**	1.67 (0.98, 2.82)	**1.87 (1.08, 3.22)**	**Oral Semaglutide**	0.80 (0.40, 1.60)
0.97 (0.67, 1.39)	0.92 (0.61, 1.39)	0.86 (0.59, 1.27)	0.83 (0.57, 1.22)	0.81 (0.55, 1.20)	0.91 (0.60, 1.37)	**0.49 (0.26, 0.91)**	**Once-weekly Semaglutide**
Network meta-analysis of severe hypoglycemia (lower left) and thyroid carcinoma (upper right)							
**Placebo**	1.00 (0.02, 50.37)	0.43 (0.11, 1.66)	0.41 (0.15, 1.18)	0.60 (0.14, 2.51)	1.00 (0.06, 15.99)	0.20 (0.01, 4.16)	2.00 (0.18, 22.08)
**1.78 (1.15, 2.77)**	**Albiglutide**	0.43 (0.01, 27.06)	0.41 (0.01, 23.92)	0.60 (0.01, 38.91)	1.00 (0.01, 121.65)	0.20 (0.00, 28.43)	2.00 (0.02, 198.39)
1.16 (0.83, 1.62)	0.65 (0.37, 1.13)	**Dulaglutide**	0.97 (0.18, 5.34)	1.40 (0.20, 10.04)	2.34 (0.11, 51.09)	0.47 (0.02, 12.97)	4.67 (0.30, 73.57)
0.88 (0.73, 1.06)	**0.49 (0.31, 0.80)**	0.76 (0.52, 1.11)	**Exenatide**	1.45 (0.25, 8.51)	2.42 (0.12, 46.72)	0.48 (0.02, 11.97)	4.83 (0.35, 66.25)
**1.35 (1.06, 1.73)**	0.76 (0.46, 1.26)	1.17 (0.77, 1.77)	**1.54 (1.13, 2.09)**	**Liraglutide**	1.67 (0.07, 37.80)	0.33 (0.01, 9.57)	3.34 (0.20, 54.66)
1.72 (0.89, 3.33)	0.96 (0.44, 2.14)	1.49 (0.71, 3.12)	1.96 (0.99, 3.89)	1.27 (0.63, 2.57)	**Lixisenatide**	0.20 (0.00, 12.19)	2.00 (0.05, 78.32)
0.56 (0.28, 1.11)	**0.31 (0.14, 0.71)**	0.48 (0.23, 1.04)	0.64 (0.31, 1.30)	**0.41 (0.20, 0.86)**	**0.33 (0.13, 0.84)**	**Oral Semaglutide**	10.02 (0.21, 481.22)
0.93 (0.79, 1.10)	**0.52 (0.33, 0.84)**	0.81 (0.55, 1.17)	1.06 (0.83, 1.36)	**0.69 (0.51, 0.93)**	0.54 (0.27, 1.07)	1.66 (0.82, 3.36)	**Once-weekly Semaglutide**
Network meta-analysis of pancreatitis (lower left)							
**Placebo**							
0.70 (0.27, 1.84)	**Albiglutide**						
0.56 (0.29, 1.11)	0.81 (0.25, 2.63)	**Dulaglutide**					
0.84 (0.48, 1.49)	1.20 (0.39, 3.69)	1.49 (0.61, 3.62)	**Exenatide**				
1.28 (0.69, 2.37)	1.83 (0.58, 5.76)	2.27 (0.90, 5.69)	1.52 (0.66, 3.52)	**Liraglutide**			
1.60 (0.52, 4.90)	2.29 (0.52, 10.04)	2.84 (0.77, 10.53)	1.90 (0.54, 6.68)	1.25 (0.35, 4.50)	**Lixisenatide**		
3.00 (0.31, 28.89)	4.29 (0.37, 50.34)	5.33 (0.50, 56.65)	3.57 (0.35, 36.85)	2.35 (0.22, 24.56)	1.87 (0.15, 23.42)	**Oral Semaglutide**	
1.33 (0.56, 3.18)	1.91 (0.52, 6.99)	2.37 (0.79, 7.13)	1.59 (0.56, 4.48)	1.04 (0.36, 3.03)	0.83 (0.20, 3.43)	0.44 (0.04, 5.02)	**Once-weekly Semaglutide**

Primary outcomes include major adverse cardiovascular events (MACE; defined as the composite endpoint of cardiovascular (CV) death, fatal or non-fatal myocardial infarction (MI) and fatal or non-fatal stroke, termed three-component MACE), fatal or non-fatal myocardial infarction, fatal or non-fatal stroke, cardiovascular death, all-cause mortality, hospital admission for heart failure (HF), severe hypoglycemia, pancreatitis, and thyroid carcinoma. Comparisons should be read from left to right. Estimates are located at the intersection of the column defining treatment and the row defining treatment. For boxes in the lower left, an OR < 1 favors the column defining treatment, while among the boxes in the upper right, an OR < 1 favors the row defining treatment. To obtain OR values for comparisons in the opposing direction, reciprocals should be taken. OR values in bold indicate statistical significance at a threshold of p < 0.05.

Fewer non-fatal and fatal myocardial infarction events were associated with albiglutide compared with lixisenatide (OR, 0.72; 95% CI: 0.55, 0.94), exenatide (OR, 0.76; 95% CI: 0.60, 0.96), and placebo (placebo *vs.* albiglutide: OR, 1.34; 95% CI: 1.10, 1.64), respectively (Table 2). Conversely, placebo was associated with a greater number of fatal or non-fatal stroke events compared with dulaglutide (OR, 1.31; 95% CI: 1.061, 1.62) and oral semaglutide (OR, 1.65; 95% CI: 1.01, 2.67). In addition, lixisenatide was associated with a greater number of fatal or non-fatal stroke events compared with semaglutide (OR, 1.84; 95% CI: 1.01, 3.35) (Table 2). A greater number of cardiovascular deaths was associated with placebo compared with liraglutide (OR, 1.29; 95% CI: 1.07, 1.54) and oral semaglutide (OR, 2.02; 95% CI: 1.08, 3.77). Moreover, lixisenatide was associated with a greater number of cardiovascular deaths compared with oral semaglutide (OR, 1.99; 95% CI: 1.03, 3.87) (Table 2).

Placebo was associated with higher all-cause mortality compared with exenatide (OR, 1.16; 95% CI: 1.02, 1.31), liraglutide (OR, 1.19; 95% CI: 1.03, 1.37), and oral semaglutide (OR, 1.98; 95% CI: 1.19, 2.29). Conversely, oral semaglutide was associated with lower all-cause mortality compared with once-weekly semaglutide (OR, 0.49; 95% CI: 0.26, 0.91), albiglutide (OR, 0.53; 95% CI: 0.31, 0.51), dulaglutide (OR, 0.56; 95% CI: 0.33, 0.95), exenatide (OR, 0.58; 95% CI: 0.35, 0.98), and lixisenatide (OR, 0.54; 95% CI: 0.31, 0.92) (Table 2).

Albiglutide was significantly less likely to induce hypoglycemia than exenatide (OR, 0.49; 95% CI: 0.31, 0.80), oral semaglutide (OR, 0.31; 95% CI: 0.14, 0.71), once-weekly semaglutide (OR, 0.52; 95% CI: 0.33, 0.84), or placebo (placebo *vs.* albiglutide: OR, 1.78; 95% CI: 1.15, 2.77), respectively. Meanwhile, liraglutide was significantly less likely to induce hypoglycemia than oral semaglutide (OR, 0.41; 95% CI: 0.20, 0.86), once-weekly semaglutide (OR, 0.69; 95% CI: 0.51, 0.93), or placebo (placebo *vs.* liraglutide: OR, 1.35; 95% CI: 1.06, 1.73), respectively. Lixisenatide was significantly less likely to induce hypoglycemia than oral semaglutide (OR, 0.33; 95% CI: 0.13, 0.84), while exenatide was more likely to induce hypoglycemia than liraglutide (OR, 1.54; 95% CI: 1.13, 2.09) (Table 2). In contrast, none of these treatments were significantly better than another in terms of hospital admission for heart failure, thyroid carcinoma, or pancreatitis (Table 2).

**FIGURE 3:**
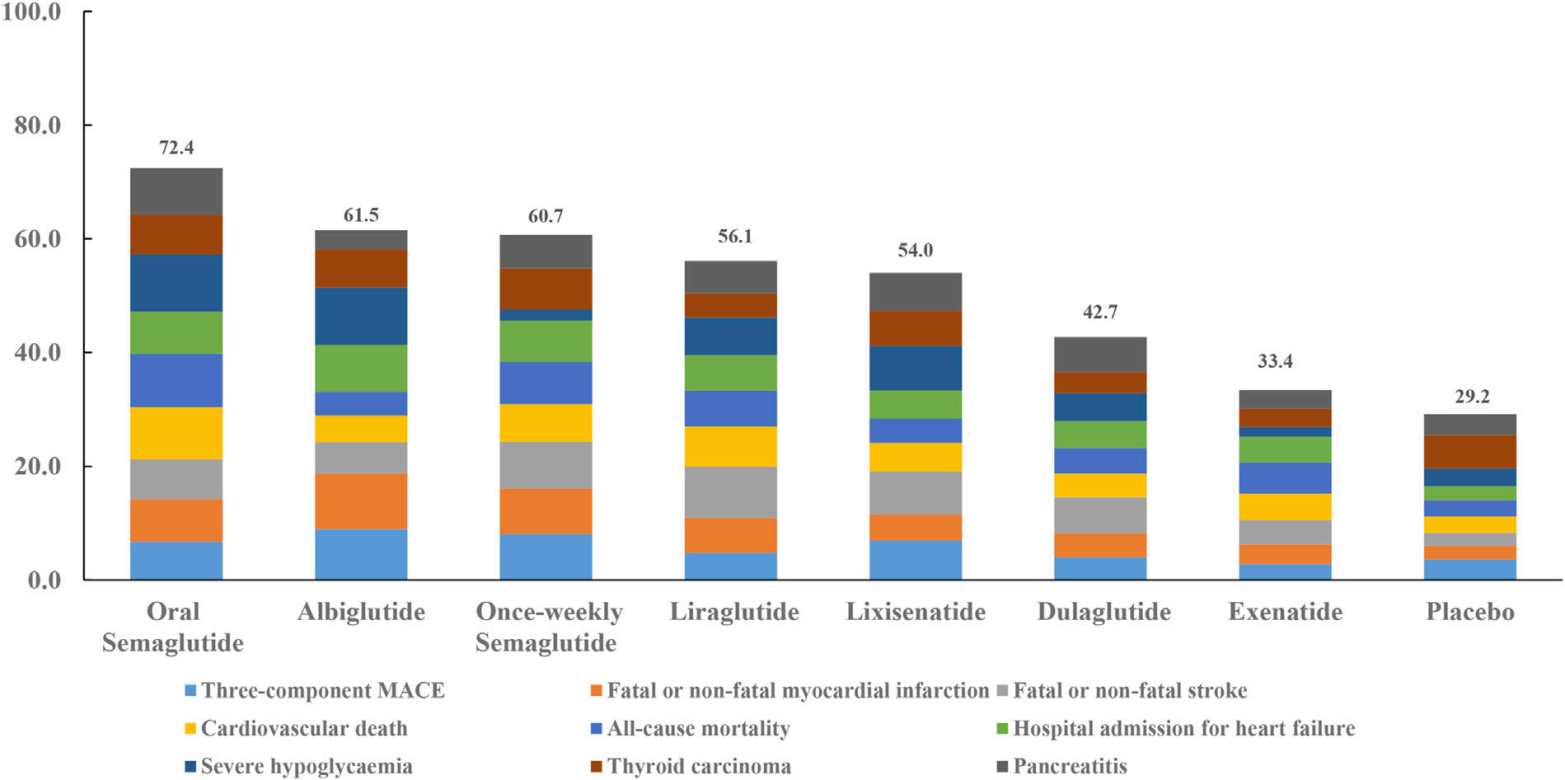
Ranking of treatments according to nine primary outcomes. Cumulative percentages after normalization (0–100%) are shown in the key. Each intervention was scored with points up to a maximum of 11.1 for each primary outcome (overall maximum score, 100) with data from rankograms and SUCRAs.

When the eight interventions were ranked according to SUCRA, albiglutide (80.6%), albiglutide (88.2%), once-weekly semaglutide (74.1%), oral semaglutide (82.8%), oral semaglutide (84.5%), albiglutide (74.2%), albiglutide (90.5%), semaglutide (65.2%), and oral semaglutide (73.4%) had the highest possibilities of being ranked first in terms of MACE, myocardial infarction, stroke, cardiovascular death, all-cause mortality, hospital admission for heart failure, severe hypoglycemia, thyroid carcinoma, and pancreatitis, respectively (Table 3). Ranking all eight interventions according to their overall probability of being ranked first after equal weighting of the nine primary outcomes identified the top four ranked interventions as: oral semaglutide (72.4%), albiglutide (61.5%), once-weekly semaglutide (60.7%), and liraglutide (56.1%), respectively (Figure 3).

**TABLE 3:** SUCRAs of treatments according to nine primary outcomes.

	Albiglutide	Dulaglutide	Exenatide	Liraglutide	Lixisenatide	Oral semaglutide	Once-weekly semaglutide	Placebo
MACE	80.6	36.1	25.2	43.2	62.7	60.9	72.8	32.9
Myocardial infarction	88.2	38.0	31.5	55.3	41.0	67.2	72.1	21.0
Stroke	49.5	57.2	38.6	38.2	68.3	62.7	74.1	21.0
Cardiovascular death	42.3	37.6	41.5	64.0	45.2	82.8	59.6	25.7
All-cause mortality	37.6	40.4	49.4	56.0	38.5	84.5	66.8	25.8
Heart failure	74.2	42.6	41.1	56.7	44.4	66.9	65.2	22.4
Severe hypoglycemia	90.5	44.0	15.0	59.2	70.3	90.2	17.6	27.5
Thyroid carcinoma	60.3	33.1	29.3	38.7	55.8	63.1	65.2	53.4
Pancreatitis	30.5	55.5	29.2	51.2	59.8	73.7	52.8	32.9
Average	61.5	42.7	33.4	56.1	54.0	72.4	60.7	29.2

Primary outcomes include: major adverse cardiovascular events (MACE), fatal or non-fatal myocardial infarction, fatal or non-fatal stroke, cardiovascular death, all-cause mortality, hospital admission for heart failure, severe hypoglycemia, pancreatitis, and thyroid carcinoma. Higher SUCRA, values are indicative of better treatments.

### Network Meta-Analyses of Secondary Outcomes

Five trials (ELIXA, EXSCEL, LEADER, REWIND, SUSTAIN-6) were included in a network meta-analysis of composite kidney outcome, worsening kidney function, and macroalbuminuria. Placebo was associated with worse composite kidney outcome compared with dulaglutide (OR, 1.18; 95% CI: 1.06, 1.30), liraglutide (OR, 1.28; 95% CI: 1.08, 1.51), and once-weekly semaglutide (OR, 1.65; 95% CI: 1.19, 2.28). In addition, dulaglutide (OR, 1.40; 95% CI: 1.00, 1.97) and exenatide (OR, 1.47; 95% CI: 1.03, 2.09) were associated with worse kidney outcome compared to once-weekly semaglutide (Table 4). Dulaglutide was also associated with fewer cases of worsening kidney function compared with lixisenatide (OR, 0.60; 95% CI: 0.36, 0.98) and placebo (OR, 0.70; 95% CI: 0.58, 0.86) (Table 4). Meanwhile, placebo was associated with a greater number of macroalbuminuria events compared with dulaglutide (OR, 1.31; 95% CI: 1.15, 1.49), liraglutide (OR, 1.35; 95%CI 1.10, 1.66), and once-weekly semaglutide (OR, 1.88; 95% CI: 1.30, 2.74). Exenatide (OR, 1.54; 95% CI: 1.00, 2.38) and lixisenatide (OR, 1.57; 95% CI: 1.02, 2.41) were associated with a greater number of macroalbuminuria events compared with once-weekly semaglutide (Table 4).

**TABLE 4:** Network meta-analysis of five secondary outcomes.

Network meta-analysis of composite kidney outcome						
**Placebo**						
**1.18 (1.06, 1.30)**	**Dulaglutide**					
1.13 (0.97, 1.30)	0.96 (0.80, 1.14)	**Exenatide**				
**1.28 (1.08, 1.51)**	1.08 (0.89, 1.32)	1.13 (0.91, 1.41)	**Liraglutide**			
1.19 (0.97, 1.47)	1.01 (0.80, 1.28)	1.06 (0.82, 1.37)	0.93 (0.71, 1.22)	**Lixisenatide**		
**1.65 (1.19, 2.28)**	**1.40 (1.00, 1.97)**	**1.47 (1.03, 2.09)**	1.29 (0.90, 1.86)	1.39 (0.94, 2.04)	**Once-weekly Semaglutide**	
Network meta-analysis of worsening kidney function						
**Placebo**						
**1.42 (1.16, 1.74)**	**Dulaglutide**					
1.11 (0.93, 1.33)	0.78 (0.60, 1.02)	**Exenatide**				
1.12 (0.83, 1.50)	0.79 (0.55, 1.12)	1.00 (0.71, 1.41)	**Liraglutide**			
0.85 (0.54, 1.34)	**0.60 (0.36, 0.98)**	0.76 (0.47, 1.24)	0.76 (0.44, 1.31)	**Lixisenatide**		
0.78 (0.38, 1.56)	0.55 (0.26, 1.13)	0.70 (0.34, 1.43)	0.69 (0.32, 1.49)	0.91 (0.39, 2.10)	**Once-weekly Semaglutide**	
Network meta-analysis of macroalbuminuria						
**Placebo**						
**1.31 (1.15, 1.49)**	**Dulaglutide**					
1.22 (0.98, 1.53)	0.94 (0.72, 1.21)	**Exenatide**				
**1.35 (1.10, 1.66)**	1.03 (0.81, 1.32)	1.10 (0.81, 1.50)	**Liraglutide**			
1.20 (0.97, 1.48)	0.92 (0.72, 1.18)	0.98 (0.72, 1.33)	0.89 (0.66, 1.19)	**Lixisenatide**		
**1.88 (1.30, 2.74)**	1.44 (0.97, 2.14)	**1.54 (1.00, 2.38)**	1.39 (0.91, 2.14)	**1.57 (1.02, 2.41)**	**Once-weekly Semaglutide**	
Network meta-analysis of retinopathy						
**Placebo**						
1.14 (0.84, 1.55)	**Albiglutide**					
0.80 (0.59, 1.08)	0.70 (0.45, 1.07)	**Dulaglutide**				
1.11 (0.92, 1.34)	0.97 (0.68, 1.39)	1.39 (0.97, 1.99)	**Exenatide**			
0.86 (0.65, 1.15)	0.76 (0.50, 1.15)	1.09 (0.72, 1.64)	0.78 (0.56, 1.09)	**Liraglutide**		
0.89 (0.67, 1.17)	0.77 (0.51, 1.17)	1.11 (0.74, 1.68)	0.80 (0.57, 1.12)	1.02 (0.69, 1.52)	**Oral Semaglutide**	
**0.57 (0.36, 0.91)**	**0.50 (0.29, 0.87)**	0.72 (0.41, 1.25)	**0.52 (0.31, 0.85)**	0.66 (0.38, 1.14)	0.65 (0.38, 1.11)	**Once-weekly Semaglutide**

The secondary outcomes included: composite kidney outcome, worsening kidney function, macroalbuminuria, and retinopathy. Comparisons should be read from left to right. Estimates are located at the intersection of the column defining treatment and the row defining treatment. For boxes colored green, an OR < 1 favors the column defining treatment. To obtain OR, values for comparisons in the opposing direction, reciprocals should be taken. OR values in bold indicate statistical significance at a threshold of p < 0.05.

**FIGURE 4:**
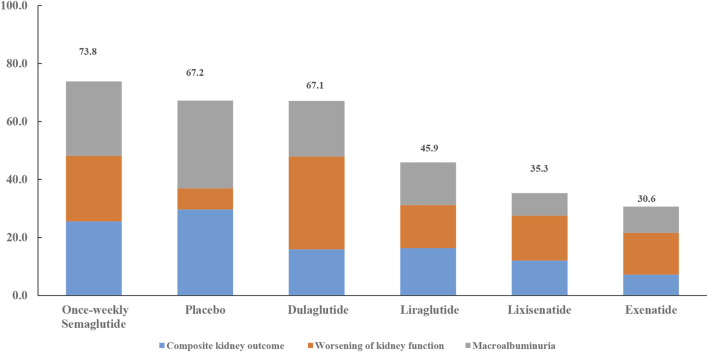
Ranking of treatments according to composite kidney outcome, worsening kidney function, and macroalbuminuria. Cumulative percentages after normalization (0–100%) are shown in the key. Each intervention was scored with points up to a maximum of 33.3 for each primary outcome (overall maximum score, 100) with data from rankograms and SUCRAs.

Except for placebo, once-weekly semaglutide, dulaglutide, and once-weekly semaglutide had the highest probabilities of being ranked first in terms of composite kidney outcome (76.6%), worsening of kidney function (95.9%), and macroalbuminuria (77.2%), respectively (Table 5). All seven interventions were ranked by their overall probability to be ranked first after equal weighting composite kidney outcome, worsening kidney function, and macroalbuminuria. The top four ranked interventions were once-weekly semaglutide (73.8%), placebo (67.2%), dulaglutide (67.1%), and liraglutide (45.9%), respectively (Figure 4).

**TABLE 5:** SUCRAs of treatments according to five secondary outcomes.

	Once-weekly semaglutide	Placebo	Dulaglutide	Liraglutide	Lixisenatide	Exenatide	
Composite kidney outcome	76.6	89	47.8	48.9	36	21.6	
Worsening kidney function	67.7	21.9	95.9	44.7	46.7	43.1	
Macroalbuminuria	77.2	90.8	57.5	44.1	23.2	27.2	
Average	73.8	67.2	67.1	45.9	35.3	30.6	
Retinopathy	Albiglutide	Exenatide	Placebo	Oral Semaglutide	Liraglutide	Dulaglutide	Once-weekly Semaglutide
	85.8	85.4	68.0	48.2	29.2	27.3	22.7

Secondary outcomes included: composite kidney outcome, worsening kidney function, macroalbuminuria, and retinopathy. Higher SUCRA, values are indicative of better treatments.

There were six trials (EXSCEL, HARMONY, LEADER, PIONEER-6, REWIND, SUSTAIN-6) which were included in a network meta-analysis of retinopathy. Albiglutide (OR, 0.50; 95% CI: 0.29, 0.87), exenatide (OR, 0.52; 95% CI: 0.31, 0.85), and placebo (OR, 0.57; 95% CI: 0.36, 0.91) was associated with fewer retinopathy events compared with once-weekly semaglutide (Table 4). Among the seven interventions, albiglutide (85.8%) had the highest probability of being ranked first (Table 5).

## Discussion

The goal of the present study was to perform a network meta-analysis which would provide unified hierarchies of evidence for all of the RCTs of GLP-1 RAs published to date. In addition to the beneficial cardiovascular effect observed among diabetic patients in our network meta-analysis, clinical data also support a protective effect of incretin therapies on cardiovascular outcomes in diabetic patients with either ST elevation myocardial infarction with culprit obstructive lesion and multivessel non-obstructive coronary stenosis (Marfella et al., 2018), or non-ST-elevation myocardial infarction with non-obstructive coronary artery stenosis (Marfella et al., 2017). Moreover, GLP-1 RA therapy plus standard hypoglycemic drugs, compared to standard hypoglycemic drugs alone, for the treatment of diabetic patients that are undergoing cardiac resynchronization therapy with a defibrillator (CRT-d) for a failing heart has lead to significant improvements in *left ventricular ejection fraction*, a reduction in New York Heart Association class, arrhythmic burden, and hospitalization for heart failure worsening, and a 3.7-fold higher rate of CRTd responders (Sardu et al., 2018).

In the present study, mitigation of albuminuria and reduced deterioration of kidney function were observed. For example, short-term liraglutide treatment decreased proximal tubular sodium reabsorption and angiotensin II concentration, thereby contributing to renal protection (Skov et al., 2016). In another study, it was demonstrated that GLP-1 can act as a physiologically natriuretic factor in the proximal tubule by modulating the activity of sodium-hydrogen exchanger isoform-3 (NHE3). As a result, GLP-1 contributes to a reduction of albuminuria through amelioration of tubule-glomerular feedback (Farah et al., 2016). Another study confirmed that administration of a GLP-1 RA increases urinary pH and urinary sodium excretion, probably due to GLP-1 RA-induced pressure natriuresis or inhibition of NHE3 in the proximal tubule (Tonneijck et al., 2016).

Regarding the beneficial effect of GLP-1 RA on risk of hypoglycemia, this effect was found to vary between studies. GLP-1 is an incretin hormone that is expressed in the gut. This hormone suppresses secretion of glucagon and stimulates production of insulin to reduce food intake and appetite and inhibit gastric emptying (Holst, 2007). However, exendin-4-based, short-acting GLP-1 RAs primarily inhibit gastric emptying to lower postprandial blood glucose levels. Meanwhile, long-acting compounds based on human GLP-1 have exhibited a stronger effect on fasting glucose levels via glucagonostatic and insulinotropic mechanisms (Meier, 2012). None of the GLP-1 RAs examined performed significantly better than the others in relation to thyroid carcinoma.

According to the overall ranking of the GLP-1 RAs examined after considering nine primary outcomes, we observed that human GLP-1 analogs achieve more pronounced effects than exendin-based GLP-1 RAs, possibly due to structural differences in the GLP-1 RA groups. While exenatide and lixisenatide are remarkably similar structurally to albiglutide, dulaglutide, exenatide, and liraglutide, there are small differences in the molecular structures of these compounds which can potentially lead to critical differences. Thus, our findings may reflect functional differences between the GLP-1 RAs or features of the trials conducted.

It should be considered that the ranking probabilities identified from this network meta-analysis are based on indirect comparisons and involve several potential limitations. For example, aggregate trial-level data were used to pool the overall estimate instead of patient-level data. Secondly, the precise exclusion and inclusion criteria of the included trials, as well as the definitions of outcomes, varied slightly. Thirdly, variations in treatment duration could have had a significant impact on overall outcomes. The median follow-up time for the COVTs compared ranged from 1.3 to 5.4 years. Fourth, the number of comparisons and the sample size for each comparison varied significantly. Consequently, a subset of the studies may have had a greater significant impact on overall effect size than anticipated, while another subset of the studies may have had less of an impact on effect size than expected. Fifth, although the proportion of patients receiving SGLT2 inhibitors and DPP-4 inhibitors was quite low, the initial usage and introduction of these treatments to patients who were receiving GLP1 therapy during the COVT trials may have affected the clinical outcomes and results of our network meta-analysis. Sixth, it should be recognized that all of the statements made which compare the merits of one GLP-1 RA with another include potential uncertainties and biases which derive from the cohorts and treatment doses studied. Finally, our analysis was limited by the small number of CVOTs conducted for GLP-1 RAs. However, despite these considerations, the suggested hierarchies of GLP-1 RAs which were elucidated in this network meta-analysis may help clinicians individualize treatment plans for T2DM patients, while also improving clinical practice guidelines.

## Conclusion

Cardio-renal benefits of GLP-1 RAs vary in patients with T2DM. Therefore, selection of GLP-1 RAs for treatment of T2DM should be individualized according to the safety profiles of the agonists considered.

## Data Availability

The original contributions presented in the study are included in the article/Supplementary Material, further inquiries can be directed to the corresponding authors.
