# Supplementary material for: Comparative Cardio-Renal Outcomes of Type 2 Diabetes Patients Administered Glucagon-Like Peptide-1 Receptor Agonists: A Network Meta-Analysis
Source: Front Pharmacol. 2021 Dec 24;12:759262. doi: 10.3389/fphar.2021.759262 (PMC8741261; doi:10.3389/fphar.2021.759262)
Supplement: Supplementary file 1 [file DataSheet1.docx]

**Supplementary Table 1.** Methodology of the studies included in the meta-analysis.

| Study | ELIXA | LEADER | SUSTAIN-6 | EXSCEL | Harmony Outcomes | REWIND | PIONEER-6 |
| --- | --- | --- | --- | --- | --- | --- | --- |
| Trial No. | NCT01147250 | NCT01179048 | NCT01720446 | NCT01144338 | NCT02465515 | NCT01394952 | NCT02692716 |
| Drug studied | Lixisenatide | Liraglutide | Semaglutide | Exenatide | Albiglutide | Dulaglutide | Semaglutide (oral) |
| Key inclusion criteria | HbA1c 5.5-11.0%, ACS within 180 days, age≥30 years | HbA1c ≥7.0%, age ≥50 years with CVD/HF/CKD or age ≥60 years with ≥1 CVD risk factor | HbA1c ≥7.0%, age ≥50 years with CVD/HF/CKD or age ≥60 years with ≥1 CVD risk factor | HbA1c 6.5-10.0%, established CVD and primary prevention, age ≥18 years | HbA1c ≥7.0%, age ≥40 years, established CVD | HbA1c ≤9.5%, ≤2 antidiabetes drugs, Age ≥50/55/60 years with CVD/subclinical vascular/risk factors | Age ≥50 years with CVD/HF/CKD or age ≥60 years with ≥1 CVD risk factor |
| Key safety exclusion criteria | Unexplained pancreatitis, multiple endocrine neoplasia syndrome | Chronic or acute pancreatitis, multiple endocrine neoplasia syndrome, calcitonin ≥50 ng/L | Chronic or acute pancreatitis, multiple endocrine neoplasia syndrome, calcitonin ≥50 ng/L | Chronic or acute pancreatitis, multiple endocrine neoplasia syndrome, calcitonin ≥40 ng/L | eGFR <30 mL/min per 1.73m2, pancreatitis, multiple endocrine neoplasia syndrome | eGFR <15 mL/min per 1.73m2, liver disease, pancreatitis, multiple endocrine neoplasia syndrome | eGFR <30 mL/min per 1.73m2, pancreatitis, multiple endocrine neoplasia syndrome |
| Glycemic management | If screening HbA1c <8.5% down-titration of insulin or sulfonylurea, DPP-4i prohibited | If screening HbA1c <8.0% down-titration of insulin, dose escalation over 2 weeks, DPP-4i prohibited | If screening HbA1c <8.0% down-titration of insulin, dose escalation over 2 weeks, DPP-4i prohibited | At physicians’ discretion according to guidelines, DPP-4i allowed | At physicians’ discretion according to local guidelines | At physicians’ discretion according to local guidelines | At physicians’ discretion according to local guidelines, DPP-4i prohibited |
| Statistical analysis | Simultaneous assessment of non- inferiority and superiority | Non-inferiority; hierarchical testing for superiority, secondary CV endpoints | Non-inferiority: superiority testing was not prespecified | Non-inferiority; hierarchical testing for superiority, secondary CV endpoints | Non-inferiority, then secondary testing for superiority, secondary CV endpoints | Superiority, secondary outcomes CV endpoints and microvascular composite | Non-inferiority, then secondary testing for superiority, secondary CV endpoints |

**Supplementary Table 2.** Definition of outcomes for the studies included in the meta-analysis.

| Study | ELIXA | LEADER | SUSTAIN-6 | EXSCEL | Harmony Outcomes | REWIND | PIONEER-6 |
| --- | --- | --- | --- | --- | --- | --- | --- |
| Trials No. | NCT01147250 | NCT01179048 | NCT01720446 | NCT01144338 | NCT02465515 | NCT01394952 | NCT02692716 |
| Drug studied | Lixisenatide | Liraglutide | Semaglutide | Exenatide | Albiglutide | Dulaglutide | Semaglutide (oral) |
| Major adverse cardiovascular events (MACE) | Death from cardiovascular causes, nonfatal myocardial infarction, nonfatal stroke, or hospitalization for unstable angina. (MACE-4, non- inferiority) | Death from cardiovascular causes, nonfatal (including silent) myocardial infarction, or nonfatal stroke (MACE-3, non-inferiority) | Death from cardiovascular causes, nonfatal myocardial infarction (including silent), or nonfatal stroke (MACE-3, non- inferiority) | Death from cardiovascular causes, nonfatal myocardial infarction, or nonfatal stroke (MACE-3, superiority) | Death from cardiovascular causes, myocardial infarction, and stroke (MACE-3, non- inferiority) | Death from cardiovascular causes or unknown causes, non-fatal myocardial infarction, non-fatal stroke (MACE-3, superiority) | Death from cardiovascular causes (including undetermined causes of death), nonfatal myocardial infarction, or nonfatal stroke (MACE-3, non- inferiority) |
| Worsening kidney function (narrow outcome) | Doubling of serum creatinine | Doubling of serum creatinine | Doubling of serum creatinine | ≥40% worsening of eGFR, end-stage kidney disease., death due to kidney disease | No outcomes reported | ≥40% worsening of eGFR | No outcomes reported |
| Composite kidney outcome including macroalbuminuria (broad outcome) | New-onset macroalbuminuria | New-onset macroalbuminuria, doubling of serum creatinine (eGFR <45 mL/min/m2), end-stage kidney disease, death due to kidney disease | New-onset macroalbuminuria, doubling of serum creatinine (eGFR <45 mL/min/m2), end-stage kidney disease, death due to kidney disease | ≥40% worsening of eGFR, end-stage kidney disease, death due to kidney disease, new- onset persistent macroalbuminuria | No outcomes reported | New-onset macroalbuminuria, ≥30% worsening of eGFR, end-stage kidney disease. | No outcomes reported |
| Severe hypoglycemia | Clinical symptoms requiring assistance from another person + BG <36mg/dL or recovery after intake of glucose/glucagon | Clinical symptoms requiring assistance from another person | Clinical symptoms requiring assistance from another person or symptomatic hypoglycemia as confirmed on plasma glucose testing (<3.1 mmol/L [56 mg/dL]) | Clinical symptoms requiring assistance from another person | Clinical symptoms requiring assistance from another person + prompt recovery after intake of glucose/glucagon | Clinical symptoms requiring assistance from another person | Clinical symptoms requiring assistance from another person |
| Pancreatitis | Adjudicated by pancreatic safety committee; no formal criteria | Two of the following: abdominal pain, elevated lipase/amylase x3 upper reference limit, imaging findings (US, CT, MRI) | Two of the following: abdominal pain, elevated lipase/amylase x3 upper reference limit, imaging findings (US, CT, MRI) | Abdominal pain + elevated lipase/amylase x3 upper reference limit (x2 if prior pancreatitis), imaging findings (US, CT, MRI) | Adjudicated by pancreatic safety committee; no formal criteria | Two of the following: abdominal pain, elevated lipase/amylase x3 upper reference limit, imaging findings (US, CT, MRI) | Not described in detail |
| Neoplasms | Only pancreatic neoplasms adjudicated; no formal criteria for pancreatic or thyroid cancer | All neoplasms captured; medullary thyroid cancer according to pathology | All neoplasms captured; medullary thyroid cancer according to pathology | All neoplasms adjudicated; no formal criteria for pancreatic or thyroid cancer | All neoplasms captured; if calcitonin >100 ng/L referral to thyroid investigation | All neoplasms captured; specific algorithm for monitoring changes in calcitonin levels | Not described in detail |
| Retinopathy | No data on retinopathy | Need for retinal photocoagulation or treatment with intravitreal agents, vitreous hemorrhage, or the onset of diabetes-related blindness | Need for retinal photocoagulation or treatment with intravitreal agents, vitreous hemorrhage, or the onset of diabetes-related blindness | Blindness due to diabetes, other diabetic eye disease | New diabetes-related blindness and procedures (laser photocoagulation or anti-VEGF treatment or vitrectomy for diabetic retinopathy/eye disease) | Photocoagulation, anti-VEGF therapy, or vitrectomy | A mix of MedDRA terms and others; trial included scheduled eye examinations |
